# Advanced Design for High-Performance and AI Chips

**DOI:** 10.1007/s40820-025-01850-w

**Published:** 2025-07-29

**Authors:** Ying Cao, Yuejiao Chen, Xi Fan, Hong Fu, Bingang Xu

**Affiliations:** 1 Nanotechnology Center, School of Fashion and Textiles, The Hong Kong Polytechnic University, Hong Kong, 999077 People’s Republic of China; 2 Department of Mathematics and Information Technology, The Education University of Hong Kong, Hong Kong, 999077 People’s Republic of China; 3 State Key Laboratory for Powder Metallurgy, Central South University, Changsha, 410083 People’s Republic of China; 4 Ningbo Institute of Materials Technology and Engineering, Chinese Academy of Sciences, Ningbo, 315201 People’s Republic of China

**Keywords:** Artificial intelligence, Advanced chips, AI chips, Design tactics, Review and perspective

## Abstract

Recent years have witnessed transformative changes brought about by artificial intelligence (AI) techniques with billions of parameters for the realization of high accuracy, proposing high demand for the advanced and AI chip to solve these AI tasks efficiently and powerfully. Rapid progress has been made in the field of advanced chips recently, such as the development of photonic computing, the advancement of the quantum processors, the boost of the biomimetic chips, and so on. Designs tactics of the advanced chips can be conducted with elaborated consideration of materials, algorithms, models, architectures, and so on. Though a few reviews present the development of the chips from their unique aspects, reviews in the view of the latest design for advanced and AI chips are few. Here, the newest development is systematically reviewed in the field of advanced chips. First, background and mechanisms are summarized, and subsequently most important considerations for co-design of the software and hardware are illustrated. Next, strategies are summed up to obtain advanced and AI chips with high excellent performance by taking the important information processing steps into consideration, after which the design thought for the advanced chips in the future is proposed. Finally, some perspectives are put forward.

**Graphical Abstract:**

## Highlights

- A comprehensive review focused on the recent advancement of the advanced and artificial intelligence (AI) chip is presented.
- The design tactics for the enhanced and AI chips can be conducted from a diversity of aspects, with materials, circuit, architecture, and packaging technique taken into considerations, for the pursuit of multimodal data processing abilities, robust reconfigurability, high energy efficiency, and enhanced computing power.
- A broad outlook on the future considerations of the advanced chip is put forward.

## Graphical abstract

**Figure:**
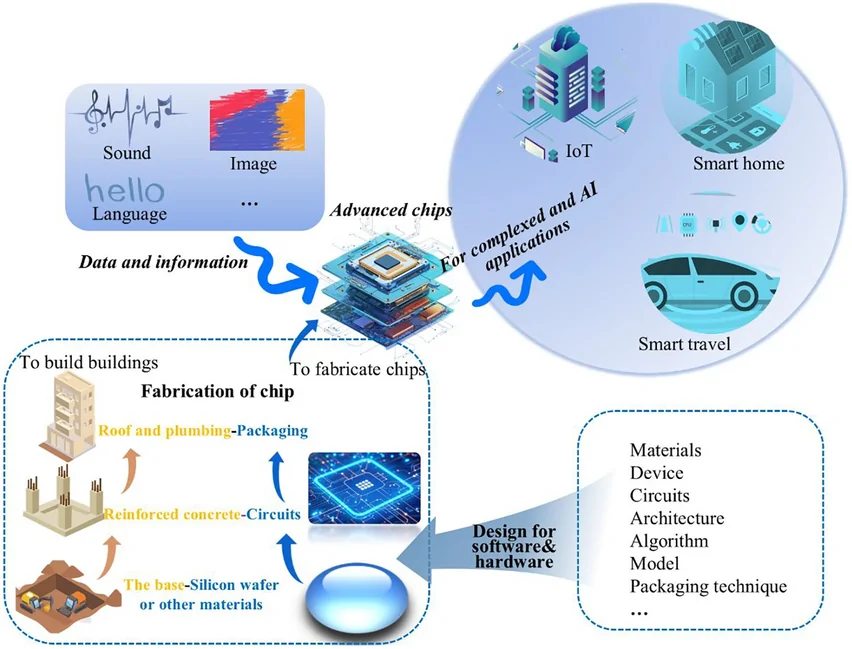

## Introduction

The past decade has witnessed the rapid progress of artificial intelligence (AI) techniques, which has revolutionized a wide range of fields, including the way to interpret information, the approach to discovery new materials, the method for creative work, and so on [1–9]. Particularly, great progress has been made in the functional materials and novel devices [10–12], which calls for AI to further promote these fields. Models of AI contain billions of parameters for the realization of high accuracy, which proposes high demand for the energy efficiency of processors. For instance, the deep neural network (DNN) model which contains many parameters can greatly promote the development of the recognition of images [13], the classification of videos, the transcription of speech [14, 15], and so on. To be specific, it has been verified that transformer and recurrent neural network transducer (RNNT) models with up to one billion parameters have shown a remarkable decrease in word error rate (WER) for the automated transcription of spoken English-language sentences. In addition to transcription, deep learning (DL) has also enhanced the performance of computer vision remarkably, which has been widely applied in the fields of autonomous driving [16], intelligent robotics [17], smart wearable devices [18, 19], and so on. Accordingly, new challenges have been put forward for the chips to handle these AI tasks. The advanced chips, which are featured with improved computing efficiency, reduced energy consumption, enhanced reliability, and excellent flexible expansion to be qualified for dealing with massive data, parallel tasks, and high concurrent requests proposed by the AI tasks, have drawn great attention, and significant progress of the advanced chips has been made by means of not only making improvements on the current silicon materials and silicon technologies, but also developing novel materials and modes [20]. For instance, data center chips, which are specifically designed for data centers, are featured with high performance and energy efficiency, and therefore, they are applied for cloud computing, AI training and inference, and big data analysis. Edge computing chips, which mainly pay attention to low latency, low power consumption, and miniaturization, have their advantages for the tasks required for real-time processing and environmental adaptability. Design thought for advanced chips referred to the process of transforming circuit structures and functions into physical layouts for the application of high-performance computing, covers wide aspects, which include but not limited to materials selection, device and circuit design, architecture optimization, and packaging technique development, and therefore, it is of importance for the rapid progress made in this field.

Many endeavors have been made to meet the challenges proposed by the AI tasks, with a lot of achievements and techniques emerging as the most promising approaches to address these issues [21–26]. For example, photonic computing makes it possible to process data faster and more energy efficiently [27]. At the meantime, the utilization of AI for optics can also improve the design and control of these optical systems [28–33]. Both the model training and inferential capability have been taken into considerations with the large-scale photonic chiplet and fully forward mode training being put forward. Computing-in-memory (CIM) which is inspired by the way in which human brain is used to process information has been put forward to resolve the von Neumann bottleneck [34]. Not only various synaptic arrays, but also efficient neuronal devices are developed. The advanced cognitive capabilities owned by the human brain have fueled a significant amount of AI research, which promote the development of sophisticated brain-inspired algorithms, as well as neuromorphic hardware with the pursuit to simulate various aspects of neural processing. Efforts have been made to develop efficient neuronal electronics. For instance, a novel dendrite function-like neuron has been developed [34]. Biocomputing, which is widely an interdisciplinary field combining biology and computer technology and uses other units instead of electrons or photons for information processing, has also emerged to address the existing issues. In addition to novel materials and new modes, improvements have also been made in areas of the conventional silicon-based chips, and more advanced preparation and packaging technology are proposed to deal with the increasing system complexity.

**Fig. 1:**
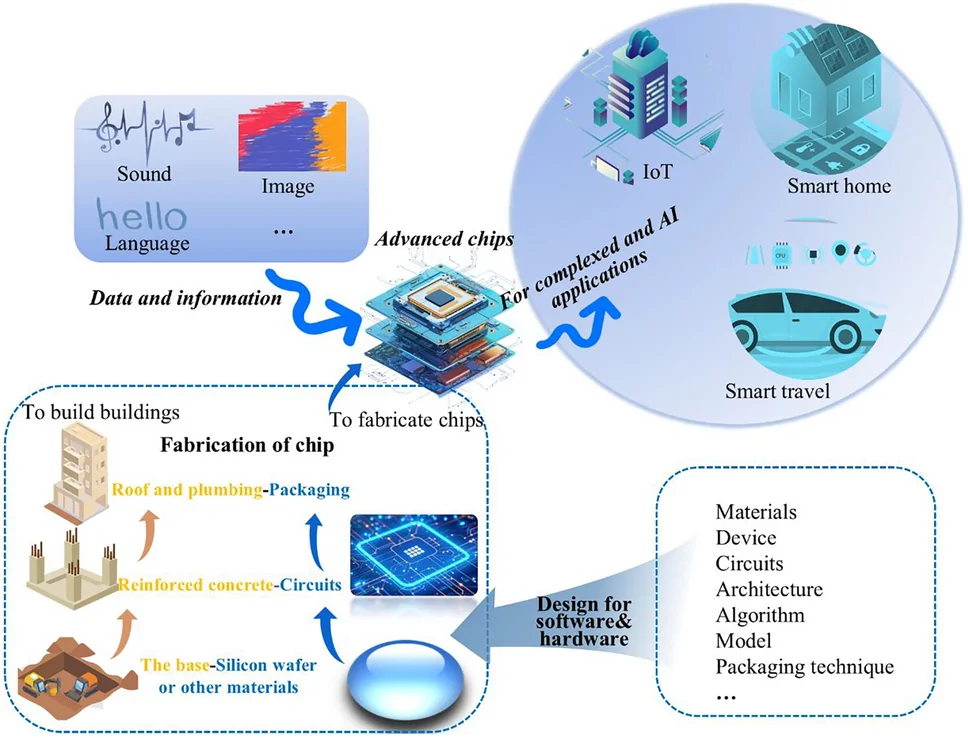
Overview of the advanced and AI chip. The design for the software and hardware favors the fabrication of the chips, which bears some analogy to the construction of buildings. The fabricated chips can then be applied to handle various information to realize complexed and AI tasks

**Fig. 2:**
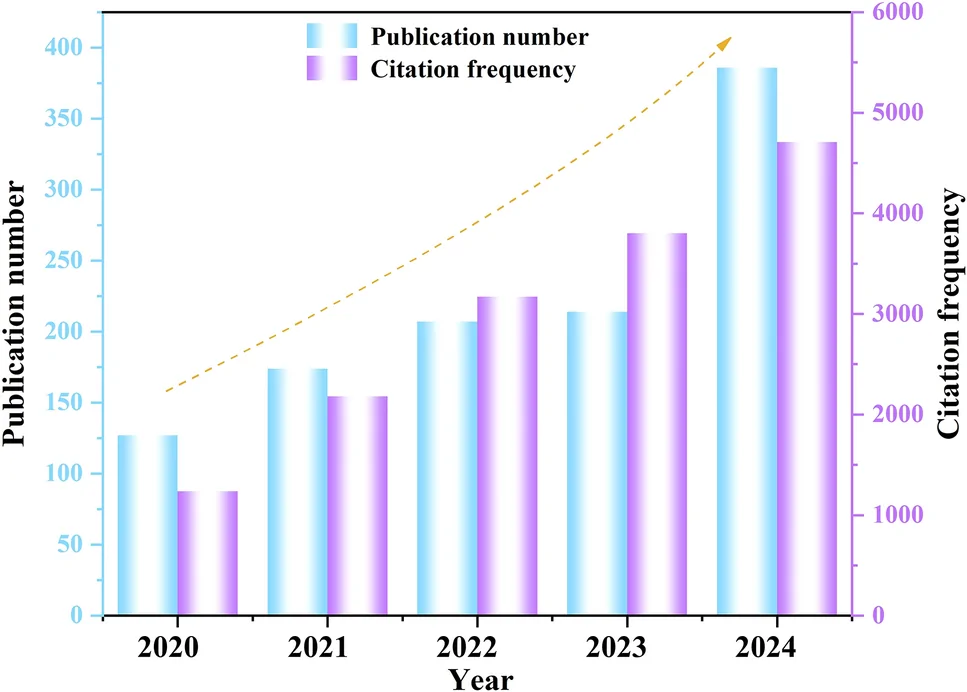
Publication and the citation frequency of the papers concerning about the AI chip. The data are collected from web of science with “AI chip” or “advanced chip” as topic words, and are also filtered according to the actual relevance of the topic

**Fig. 3:**
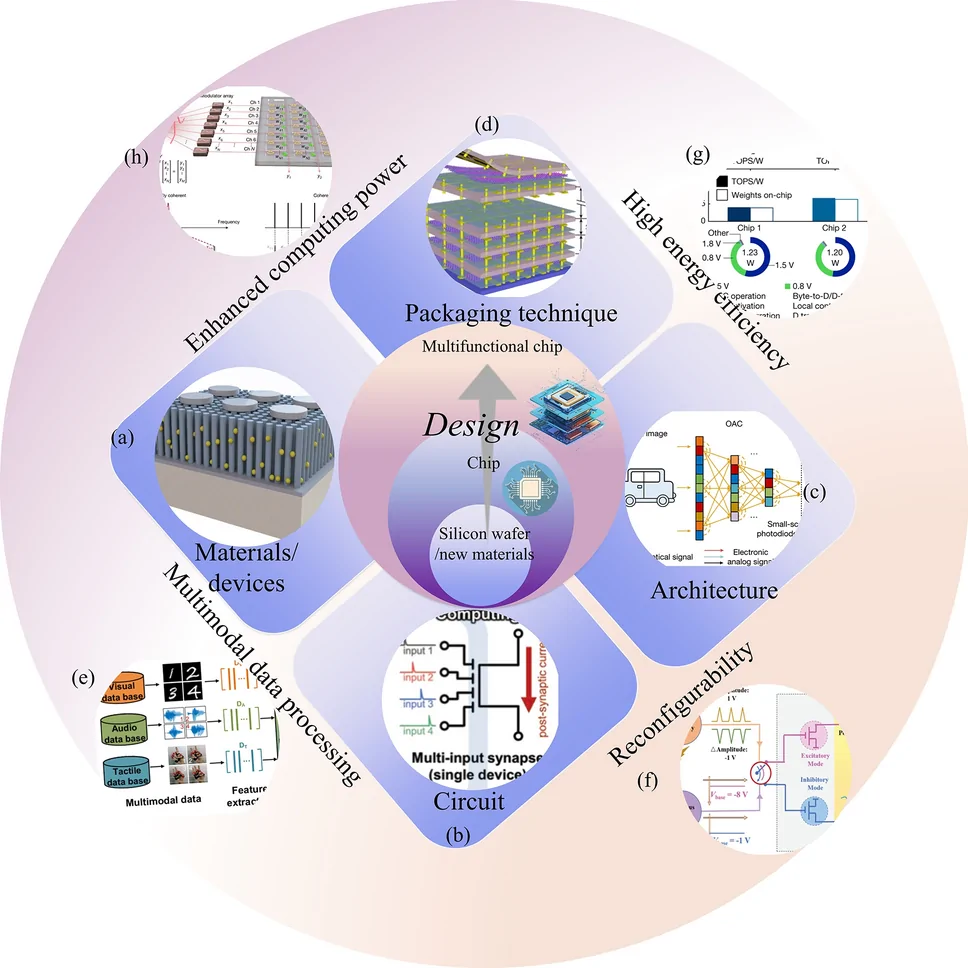
Design strategies about the advanced chips. Design strategies carried for **a** material/device, reproduced with permission from Ref. [36] Copyright 2024, Springer, **b** circuit, reproduced with permission from Ref [39]. Copyright 2024, Wiley–VCH GmbH, **c** architecture, reproduced with permission from Ref. [27] Copyright 2023, Nature, and **d** packaging technique, reproduced with permission from Ref. [40] Copyright 2024, Nature. The design objective of realizing **e** multimodal data processing, reproduced with permission from Ref [41]. Copyright 2024, Nature, **f** reconfigurability, reproduced with permission from Ref [42]. Copyright 2023, Wiley–VCH GmbH, **g** high energy efficiency, reproduced with permission from Ref. [1] Copyright 2023, Nature, and **h** enhanced computing power, reproduced with permission from Ref. [43] Copyright 2024, Nature

Significant progress has been made in both the hardware and the software of the advanced chips recently, which favors the fabrication of the chips. It is proposed that the fabrication of the chip bears some analogy to the construction of buildings. The fabricated chips can then be applied to handle various information to realize complexed and AI tasks, including computer vision, speech recognition and transcription, parallel imaging and all-optical classification, patients’ gaits classification, and other various fields, with Internet of Things (IoT), smart travel, smart robot, and smart home included (Fig. 1). An analog-AI chip with 35 million phase-change memory (PCM) devices has been developed [1]. A systemic energy efficiency of 74.8 peta-operations per second per watt is managed to be achieved by a type of all-analog photoelectronic chip [27]. Further to the inference chip, a fully forward mode (FFM) learning has been proposed for the training of optical neural networks, which is able to accomplish the compute-intensive training process on the physical system [35]. The fully hardware implementation of CIM has been experimentally realized by integrating neuron devices with a low accuracy loss [34]. Neuromorphic hardware equipped with associative learning abilities has been fabricated [36]. The low processor resting power of 0.42 mW has been achieved by a neuromorphic system on chip with the features of no-input calling for no energy, while a real-time power of as low as 0.70 mW can be realized for this system by the co-design of algorithm, software, and hardware [37]. The large-scale photonic chiplets, Taichi, which has millions-of-neurons capability with 160-tera-operations per second per watt (TOPS/W) energy efficiency, have been put forward. It has been verified that the high-fidelity AI-generated content can be realized by the photonic chiplet with up to two orders of magnitude of improvement in efficiency [38]. Publication number and the citation frequency of the papers concerning about the AI chip are counted from web of science. The data are collected with “AI chip” or “advanced chip” as topic words and are also filtered according to the actual relevance of the topic. As a result, an increasing number of original works have been published with high impact and sharply increasing citation frequency, which is demonstrated in Fig. 2. These results show that the research focused on the advanced chips has drawn great attention. The design strategies have been launched from various aspects, including materials, devices, circuits, architecture, and packaging techniques with the pursuit for multimodal data processing, reconfigurability, enhanced computing power, and high energy efficiency (Fig. 3). For instance, for multimodal data processing, which is required to handle different types of data, like images, sounds, and texts, proper packaging technology can facilitate the integration of different processing units more closely to enhance the processing speed, while reducing latency. Besides, the reconfigurable architecture which makes it possible for the hardware structure to be reconfigured according to different tasks also makes contribution to the multimodal data processing with the adjustment to different algorithm. However, reviews from the view of recent design tactics for AI chips are few. Herein, this review focused on the advanced design of the high-performance chips by means of not only making improvements on the current silicon materials and silicon technologies, but also developing novel materials and modes, like photonic computing, and the quantum processors, among which many can meet the challenges proposed by the rapidly developing AI technology.

In this review, the basic background of AI chips was introduced first, as well as their working mechanisms, after which the design ideas in regard to software and hardware from the aspects of both the technique development for the conventional silicon-based chips, and the adoption of novel modes that extend the information processing from electrons, to photons, quantum, and biological elements, were demonstrated. Key factors which should be under consideration when designing the advanced chips were discussed from the view of the information processing procedures. Last but not least, we put forward some ideas with respect to the outlook of the advanced chips.

## Mechanisms

**Fig. 4:**
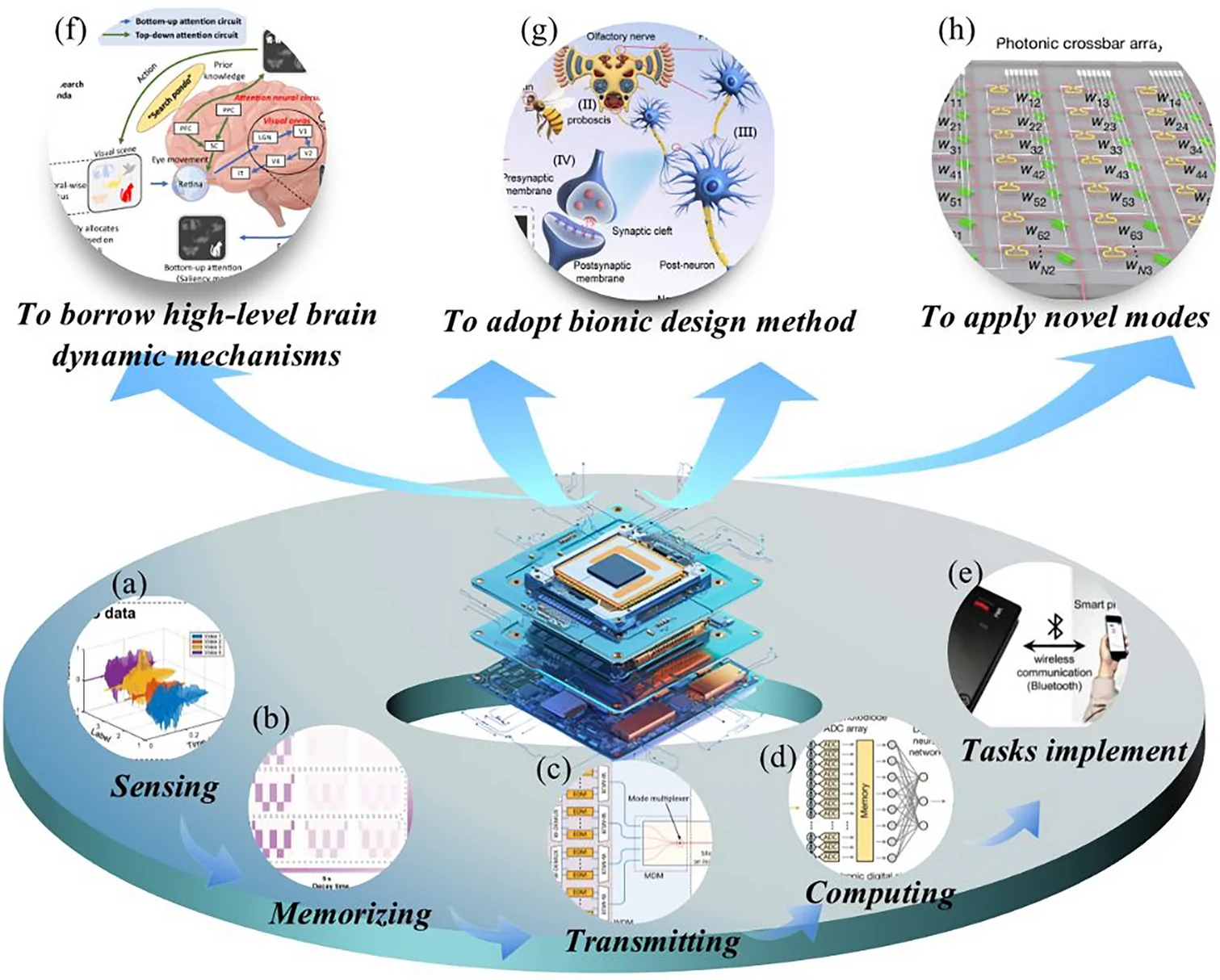
Schematic illustration for the working mechanism of the advanced chips. Schematic illustration for the stage of **a** sensing, reproduced with permission from Ref [41]. Copyright 2024, Nature, **b** memorizing, reproduced with permission from Ref. [36] Copyright 2024, Springer, **c** transmitting, reproduced with permission from Ref. [59] Copyright 2022, Nature, **d** computing, reproduced with permission from Ref. [27] Copyright 2023, Nature, and **e** task implement, reproduced with permission from Ref [39]. Copyright 2024, Wiley–VCH GmbH. Schematic illustration for the method to improve the performance of chips by **f** borrowing high-level brain dynamic mechanisms, reproduced with permission from Ref. [37] Copyright 2024, Nature, **g** adopting bionic Design method, reproduced with permission from Ref. [36] Copyright 2024, Springer, and **h** applying novel modes, reproduced with permission from Ref. [43] Copyright 2024, Nature

The chips are applied to deal with various information and data. For instance, data can be collected from multimodal sensors. As for a typical task, the information is first captured by the sensors and is then digitized by a large number of analog-to-digital converters (ADCs) [27] (Fig. 4a). Data are then processed and transmitted (Fig. 4b, c). The neural network (NN) on a digital processing unit can then be made use of to process the information for recognition, classification, and other purposes. Edge computing can implement data processing at the sensors. In particularly, as to a sensing-computing system on chip (SoC), the sensors can be integrated onto the chips to provide the information to be processed. For example, by leveraging the DVS as the eye of the chip, an asynchronous chip can be designed [44–46]. As the brightness of the scene changes, the DVS is managed to generate a stream of events asynchronously and sparsely, which can then be processed by the operation of the processor in the chip. However, it is proposed that not all sensors are solid state due to the diverse types of sensors, and therefore some are not suitable for integrated computing units. In addition to the sensing-computing system, there is also a high demand for large language model (LLM) acceleration, and therefore, how to provide strong computing power support should be taken into considerations.

The neuromorphic hardware learning from the information processing of human brain is a promising candidate for next-generation computer architectures because of its massive parallelism, robust fault tolerance, and high efficiency, which is different to the conventional architecture. The exploiting of the neuromorphic computing systems makes it possible to implement the parallel processing, which enables the execution of separate complex tasks by making use of several processors simultaneously, leading to the enhanced processing efficiency [39, 47–50]. Moreover, it is also expected for the neuromorphic systems to accomplish the processing of integrated signals from various inputs. The development of materials has promoted the realization of these functions greatly. The electrochemical artificial synapses can facilitate the simultaneous processing of multi-input signals via a unit device. The working mechanisms of the electrochemical artificial synapses composed of the electrolyte-based dielectric and ion-permeable semiconducting layer origin from the resistance tuning of the channel with penetrated ions and the retentive relaxation property.

Information is expected to be processed by the chips as the way of human brain, including learning, reasoning, and memorizing [39]. It turns out that human brain is managed to run even more complex neural networks with a total energy need of only 20 W [51, 52]. A variety of behaviors in the biological synapses, which are responsible for the information transmission between biological neurons, are simulated by the artificial neuromorphic electronics to handle the information collected by the sensors. Inspirations are also expected to be obtained from some high-level brain dynamic mechanisms in regard to the design of neuromorphic chips [53]. For the human brain, an important feature is to allocate its resources dynamically according to the required demand. To be specific, the salient stimuli can receive greater attention, which can be manifested by the heightened spiking activity in brain regions or the corresponding neurons associated with the stimulus. This high-level dynamic computing nature of the human brain is expected to be learned by the neuromorphic chips which are featured with minimal energy consumption for no input and significant variations for input changes. From the perspective of functional materials, some potential candidates, like two-terminal memristors which are featured with their compact synapse-like structures, have been extensively explored to equip the electronics with high complexity and improved completeness like the biological neurons for information transmission and processing [54, 55].

High capacity and high-throughput computing architectures are then required to handle the complex multimodality information collected from the environment [56] (Fig. 4d), and finally, the chips can be applied to implement various tasks (Fig. 4e). Great endeavors have been made to enhance these processes to improve the overall performance of the whole systems by a series of attempts, including borrowing high-level brain dynamic mechanisms (Fig. 4f), adopting bionic design approach (Fig. 4g), applying novel modes (Fig. 4h), and so on. Photonic processors are proposed to be a key to the hardware-based AI accelerators [23, 57, 58]. For the realization of in-memory photonic convolutional processing free of data movement between the memory and photonic processors, photonic tensor core incorporating phase-change-material photonic memories has been made use of. Generally, the data carried by each input coherent light at different wavelengths are weighted by the phase-change-material photonic memories. As a result, various tasks can be accomplished by the chips, ranging from computer vision, speech recognition, to gaits classification, which makes them to be qualified for a diversity of fields, including  IoTs, smart homes, intelligent robotics, and so on.

## Co-design of the Software and Hardware

AI relies on hardware and software to simulate human intelligence, and it is critical to carry out the co-design of both the software and the hardware for the advanced and AI chips. Specifically, software programming is of importance for the construction and training of  NN, while hardware is crucial to process and handle the data for AI operation [60–62]. For example, although a highly programmable accelerator architecture for analog-AI has been proposed, it has yet to be demonstrated in hardware for the reason that the simulation study contains several design assumptions, among which one is the application of a dense and efficient circuit-switched 2D mesh for the exchange of massively parallel vectors of neuron-activation data over short distances, and another is the successful realization of DNN models which are large enough to be relevant for the commercial applications while maintaining high accuracy [1]. As a result, these issues should be solved for the design and fabrication of the analog-AI chips. Another case in point is that efforts have been made to design the CIM-based hardware systems in accordance with the requirements of the AI algorithm to successfully implement the extensive tasks of AI, promoting the commercial production of the CIM-based chips [34]. In this case, elaborate designs are essential in terms of both the optimized algorithms and innovative hardware for the neuromorphic computing systems. Besides, an algorithm-software-hardware co-design has also been put forward to realize the spike-based dynamic computing in the neuromorphic chip, with the hardware featured with no running energy for no-input, and the complete software toolchain for the efficient deployment of algorithms in a variety of dynamic vision applications [37].

### Software

#### Some General Principles for Software Design

AI algorithms have been evolved rapidly. The intricate cognitive capabilities achieved by the human brain have sparked extensive research in AI with the promotion of sophisticated brain-inspired algorithms. It is worthwhile mentioning that the device-algorithm co-optimizations need to be carried out for the real-world application. Particularly, the software toolchain with data management, model simulation, and host management included is beneficial to deploy the algorithms and models efficiently for various applications [37]. Moreover, when developing different chips, the challenges and solutions at the software level are various, and design of the software is of important for all of these techniques, which lies in the aspects of model, algorithm adaptation, and toolchain. For instance, as to memristor, the integrated memory and computing architecture is required, while optical path programming is essential for photonic computing.

#### To Collaborate with the Hardware

**Fig. 5:**
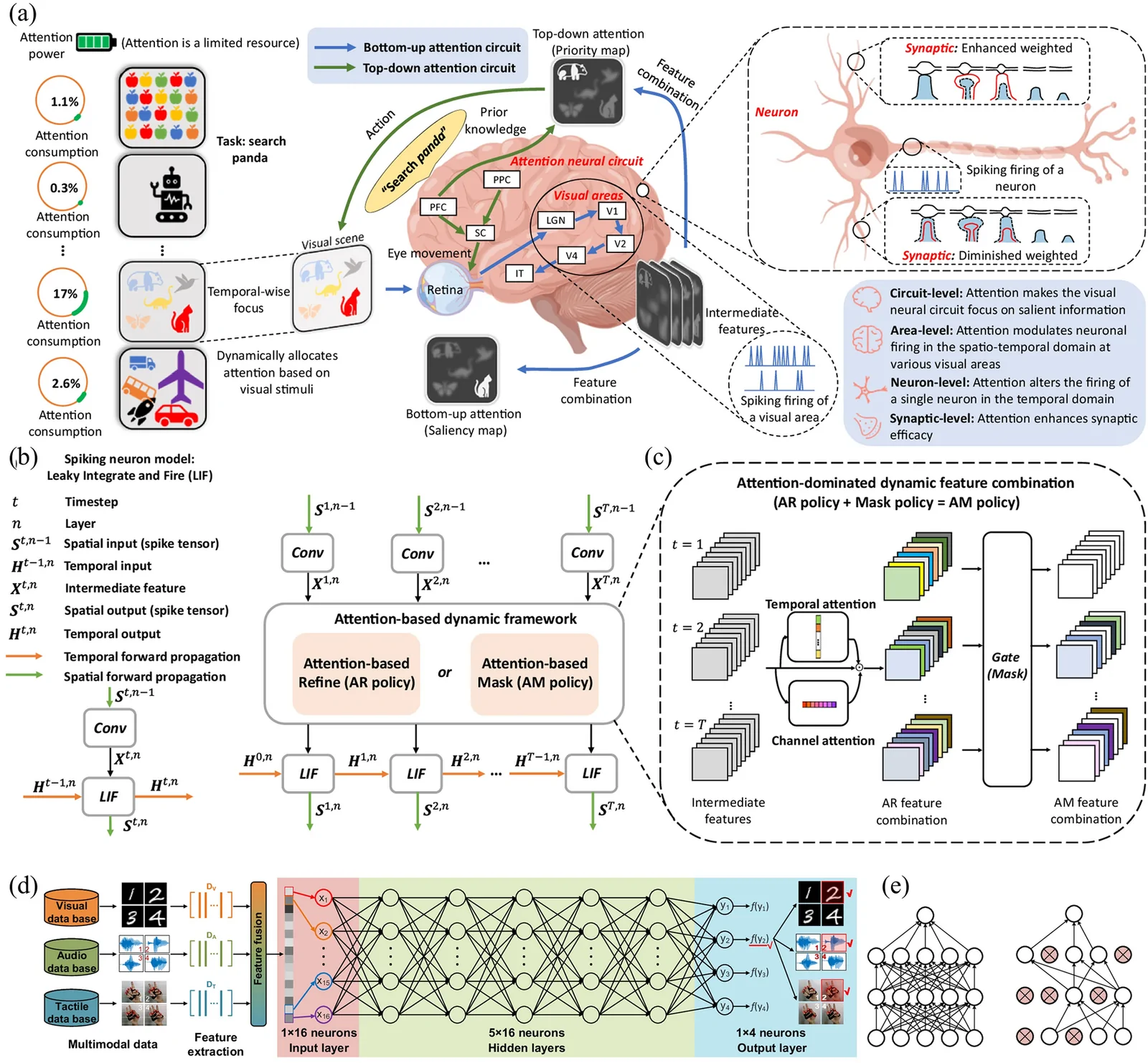
Schematic of how software designs facilitate the development of advanced chips. **a** Schematic diagram for the attention-based dynamic response in neuroscience. Illustration for **b** a typical spiking neuron model and **c** attention-based dynamic SNNs. **a**–**c** Reproduced with permission from Ref. [37] Copyright 2024, Nature. **d** Schematic diagram of the optical neural network model for multimodal classification. **e** Schematic diagram of the drop-out algorithm. **d**–**e** Reproduced with permission from Ref [41]. Copyright 2024, Nature

The design of the software plays a crucial role in achieving various advantages of the advanced chips by working together with the hardware [37]. For instance, endeavors were made to combine the high-level dynamic computing nature of the brain with machine intelligence to equip the neuromorphic computing with energy advantages. The hardware was developed to meet the demand from dynamic computing, which indicated that no-input consumed no energy. Meanwhile, the design for an attention-based framework was also carried out to meet the challenge of dynamic computing which was featured with the fact that varied inputs consumed the energy with large variance. To accomplish this goal, inspirations for designing the dynamic spiking neural networks (SNNs) were gained from the understandings of visual attention in neuroscience. To be specific, since attention is a limited resource, the brain only processes a part of sensory input selectively. The neural related to attention can be divided into four structural levels, including circuit level, area level, neuron level, and synaptic level, and a general classification of attention neural circuits is the top-down versus bottom-up dichotomy (Fig. 5a). Top-down allocates the attention to internal behavioral goals of the brain, which can be presented through the priority map, while bottom-up deploys attention corresponding to the physical salience of a stimulus. As for the design of the framework for neuromorphic computing, a typical spiking neuron model and attention-based dynamic SNNs were illustrated as Fig. 5b, c. It was worthwhile mentioning that the dynamic framework acted as plug-and-play attention modules with the membrane potential optimized in a data-dependent manner, and combinable strategies of refinement and masking were provided by this dynamic framework. It was verified that a real-time power as low as 0.70 mW was successfully achieved by this neuromorphic system.

#### To Conduct the Design of Algorithm

Some challenges brought by the explosive growth of the AI can be met by the design of algorithm, like the issue that multiple types of data are needed to be handled along with the boost development of the artificial intelligence generated content (AIGC) [63–66]. For example, it was pointed out that the majority of photonic neuromorphic processors for DL were able to handle only a single data modality for the reason that abundant parameters for training in optical domain were lack. To address this issue, a trainable diffractive optical neural network (TDONN) chip weas developed. In particular, the optical neural network model designed for the multimodal classification tasks was formed by three parts with an input layer, five hidden layers, and an output layer included (Fig. 5d). After the procedures of feature extraction and feature fusion, a feature vector was got from the datasets of different modalities, which was then applied as the input of the NN with the size of the feature vector matching the number of neurons. Each of the vector element was encoded into the optical signal by intensity modulation. In the hidden layers, the neurons were arranged in accordance with a multi-layer layout. The connection weights between each neuron were adjusted during training, and therefore trainable neurons were deemed as a critical prerequisite for reconfigurable TDONN, since the strong reconfigurability was essential for the multimodal DL. It took two steps for training of the TDONN chip, with the first one to extract the features and the second step to train the tunable diffractive units to accomplish the target tasks. It was worthwhile mentioning that customized gradient descent algorithm and drop-out mechanism of optical neurons were designed for the realization of the function. Firstly, an iteration threshold Titer was set for each neuron in the hidden layer of TDONN. During the iteration process, for the condition where the neuron could not increase CF after T adjustments, the neuron was set to be inactivated, and in the following iterations, this inactivated neuron would not be adjusted. As the training progresses, the number of deactivated neurons increased, and only the activated neurons needed to be tuned, leading to the reduce of the workload (Fig. 5e).

### Hardware

#### Some General Principles for Hardware Design

Hardware design is imperative for promoting the development of different types of chips, the reason that it can solve the problems of different chips, making full use of these chips in various fields. To be specific, memristor, which can simulate the plasticity of biological synapses, plays a critical role in the brain-inspired computing. Photonic computing is featured with ultra high-speed, while it is also encountered with the problem of poor compatibility with silicon-based electronic chip. The computing power of quantum computing to deal with specific problems far exceeds that of classical computers, but the extremely low-temperature requirement is usually a challenge. Neuromorphic computing is managed to mimic the structure of human brain, and it can realize event-driven computing by means of asynchronous SNN, which is qualified for real-time perception and IoT. Accordingly, new circuit layout or material structure design is carried out to meet these challenges.

#### To Develop the Materials

The development of materials is served as one of the most important supports for the thriving chip industry. For instance, CIM-based hardware systems are designed according to the requirements from AI algorithm to accelerate the extensive computations by means of eliminating frequent data transfers between memory and processing units [67–69]. Accordingly, many endeavors have been made on the development of non-volatile memories (eNVMs) for the purpose of storing the weights in neural networks, with the PCM, RRAM, ferroelectric field effect transistor (FeFET), and other eNVMs included. Besides, more advanced functions are expected to be realized with high-efficiency algorithm while maintaining low hardware costs and high flexibility for the accomplishment of different application scenarios. As for the design of the hardware, a series of factors, like the stability, uniformity, and feasibility for large-scale realization, should be taken into consideration. Accordingly, efforts have been made not only by adopting novel modes, like the neuromorphic computing, photonic computing, and quantum computing, but also by improving the existing silicon chips, like the development of the package technique.

#### To Exploit New Mode: Neuromorphic Computing

Much efforts have been made on mapping the biological behavior in the nervous system to the electrical behavior in various devices, and many techniques have been emerged as the most promising approaches to meet the challenges brought by the AI tasks. It turns out that excessive energy consumption occurs with a significant amount of data moving between memory and processor, which is known as the von Neumann bottleneck [1]. CIM is proposed to be a promising approach to meet the challenge of increasing computational tasks brought about by the rapidly booming AI [34]. For the DNN models containing many large fully connected (FC) layers for the natural language processing (NLP), enormous movements of data are required in conventional digital implementation, while amortization over the subsequent computing is lacking. Analog-AI hardware is managed to meet this challenge by means of leveraging arrays of non-volatile memory (NVM) to perform the multiply–accumulate (MAC) operations, so that these workloads can be dominated directly in the memory [70–73]. When neuron-excitation data are moved to the location of the weight data, where the computation is executed, both the time and the energy are promising to be reduced. When taking the finite endurance and the power-hungry programming of NVM devices into consideration, it is inevitable that such analog-AI systems should be fully weight stationary. A highly heterogeneous and programmable accelerator architecture for analog-AI has been developed with the energy efficiencies 40–140 times higher than those of cutting-edge graphics processing units, but it has yet to be demonstrated in hardware due to the fact that several design assumptions are included [74].

**Fig. 6:**
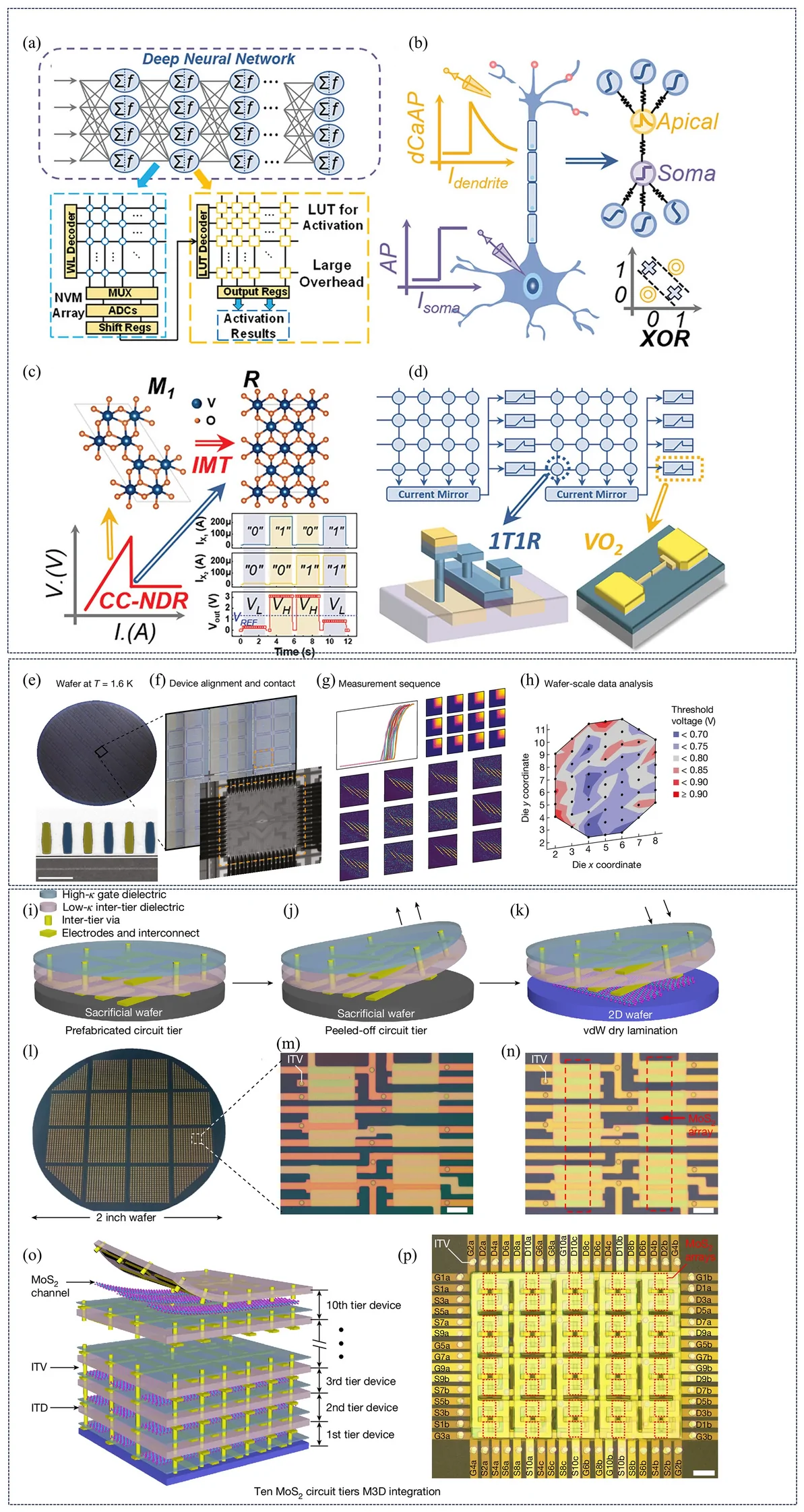
Schematic of how hardware design promotes the development of different types of chips. **a** Schematic of the DNN structure and how to be realized by conventional hardware. **b** Schematic illustration of the calcium-mediated dendritic action potentials (dCaAPs) and the conventional all-or-none APs. **c** Schematic of NDR, insulator–metal transition (IMT), and the XOR operation realized in a single device. **d** Schematic illustration for the fully-hardware implementation of DNN. **a–d** Reproduced with permission from Ref. [34] Copyright 2024, Wiley–VCH GmbH. **e** Optical image of the completed spin qubit wafer. **f** Schematic of the device alignment and contact. **g** Various measurements used to extract the data. **h** The data used for statistical analysis. **e–h** Reproduced with permission from Ref. [100] Copyright 2024, Nature. **i** Circuit tier prefabrication on a sacrificial substrate. **j** Physically peeling off circuit tier, and **k** van der Waals dry lamination. **l** Optical images and **m** the zoomed-in image of prefabricated circuit tier on 2 inch sacrificial substrate. **n** Optical image of the final device. **o** Schematic diagram and **p** optical image of a 10-tier M3D system. **i–p** Reproduced with permission from Ref. [40] Copyright 2024, Nature

Although the rapid progress has been made in CIM technology, it is crucial to recognize that the majority of the non-linear computations for the results after linear matrix–vector multiplying relies on conventional complementary metal oxide semiconductor (CMOS) circuits, with ADCs and digital circuits for complex arithmetic included, leading to excessive area and energy costs [75, 76] (Fig. 6a). It is crucial to make exploration for hardware implementation of activation functions on the basis of emerging devices and functional materials. Inspiration was obtained from dendritic computation of the pyramid neurons in the brain cortex to deal with the overhead in the hardware implementation of activation functions [34]. The distinguished calcium-mediated dendritic action potentials (dCaAPs) were brought into focus of the researchers which were in the pyramid neurons of the human layer 2 and 3 cortex. When compared to conventional all-or-none action potentials (APs), it was observed that the amplitude of dCaAPs becomes maximal for a certain threshold-level stimuli and was dampened for stronger stimuli (Fig. 6b), and therefore it was proposed that this distinctive dCaAP made it possible for a single neuron to implement XOR classification which typically required multilayered neural networks because of its inherent linear non-separability. It was pointed out that the electronic elements featured with negative differential resistance (NDR) were promising candidates of such mimicry, for which the measured response decreased as the stimulus intensity increased (Fig. 6c). NDR characteristics could be found in a wide range of electronics, among which Mott materials were one of the best candidates. As a well-studied Mott material, vanadium oxide (VO_2_) was investigated as a potential substitute for conventional activation units of NN. Moreover, this novel activation unit was managed to be integrated within a non-von Neumann architecture, which was verified by co-implementing 1T1R arrays and these neurons on a single hardware platform (Fig. 6d).

In addition to the imitating of the essential synaptic functions, the in-depth study of the underlying learning and memory mechanisms in the biological brain is also vital for the realization of intelligent information processing at the hardware level [77]. For instance, it is proposed that the hardware realization of associative learning makes contribution to improving the functionality of NN, enhancing the performance of machine learning (ML) algorithms [78, 79]. Furthermore, it can also promote the development of more autonomous machines which are featured with the ability to adapt and learn in dynamical environments without the requirement for pre-programming [80–82].

#### To Exploit New Mode: Photonic Computing

In the post-Moore era, greater challenges have been proposed for the continuous demand of higher performance [38]. Photonic computing has offered significant advantages for the unprecedented light-speed and low-consumption computing [21, 22], which empowers much faster and more energy-efficient processing of data. In this case, the features of light are made use of to represent the information, and propagation and interference are taken advantaged of for computing [57, 83–95]. Meanwhile, the utilization of AI for optics can promote the design and control of optical systems. Recently, both the photons and the electrons have been used in an all-analog way to come up with a practical solution for the intelligent computing [27]. Moreover, the development of integrated photonics also makes contribution for the implementation of intelligent tasks by the photonic computing chips [25, 96–99].

#### To Exploit New Mode: Quantum Computing

In addition to the neuromorphic computing and photonic computing, quantum computing has been emerged as another advanced type of computing [100]. To promote the applications of spin qubit technology, physical qubit count is required to increase substantially, which makes it essential to fabricate spin qubit devices with the density, volume, and uniformity comparable with those of classical computing chips composed of billions of transistors [101]. The spin qubit technology is featured with its inherent advantages for scaling due to the qubit size, and another advantage is the native compatibility with CMOS manufacturing infrastructure. As a result, it is pointed out that manufacturing spin qubit devices with the same infrastructure as classical computing chips is managed to release the potential of spin qubits for scaling, and it is possible for them to offer an approach for building the fault-tolerant quantum computers. Furthermore, the scale of cryogenic device testing must be launched to enable efficient device screening [102, 103]. Spin qubits based on electrons in Si have demonstrated impressive control fidelities, but the challenges exist in the aspects of yield and process variation. Recently, some progress has been made to address this issue. One case in point was that a testing technique taking advantages of the cryogenic 300-mm wafer prober for collecting the data in high volume on the performance of hundreds of industry-manufactured spin qubit devices at 1.6 K was developed. It took about 2 h to cool 300-mm wafers to an electron temperature of 1.6 K [100], and the transmission electron micrograph of a Si/SiGe quantum dot qubit device cross section is shown in Fig. 6e. As is demonstrated in Fig. 6f, the device pads were then aligned to the probe pins, and devices were connected to measurement electronics at room temperature. A diversity of measurements could then be used to extract the data (Fig. 6g), and when this process on many devices across the wafer was repeated, the statistical analysis of wafer-scale trends was managed to be implemented by making use of the device data, which is illustrated in Fig. 6h.

#### To Promote the Integrating Technique

Besides the new materials and novel modes for the development of the advanced chips, progress has also been made in the aspect of integrating technique [40]. Monolithic three-dimensional (M3D) integration, for which multiple stacked tiers are fabricated sequentially on the same wafer by deposition of the upper tiers, has been proposed to overcome the scaling limitation with higher device density, and it enables new 3D computation systems, in which case various tiers, like the logic, memory, and sensor, are managed to be vertically interconnected [104–106]. As to the silicon-based M3D integration, challenges exist in the aspect of the low thermal budget, for which the process temperature of upper tiers should not exceed the back-end-of-line temperature to get rid of the performance degradation. It has been pointed out that two-dimensional (2D) semiconductors are promising for M3D integration, which is attributed to their dangling-bonds-free surface and the ability to be integrated to various substrates [107–113]. Recently, an alternative low-temperature M3D integration method by van der Waals lamination of entire prefabricated circuit tiers has been developed. The detailed integration processes included the procedures of circuit tier prefabrication on a sacrificial substrate, physically peeling off circuit tier and van der Waals dry lamination, which is demonstrated in Fig. 6i–k. It was noticeable that the prefabrication of all circuit stacks was based on standard photolithography processes, and it was compatible with wafer-scale M3D integration, which is demonstrated in Fig. 6l–n. A 10-tier M3D circuit within a total thickness of approximately 8 μm could be realized to verify the high-density M3D systems with multiple circuit tiers in the vertical direction, which is shown in Fig. 6o, p.

## Strategies to Design Advanced and AI Chip with Enhanced Overall Performance

### For Memory Purpose

**Fig. 7:**
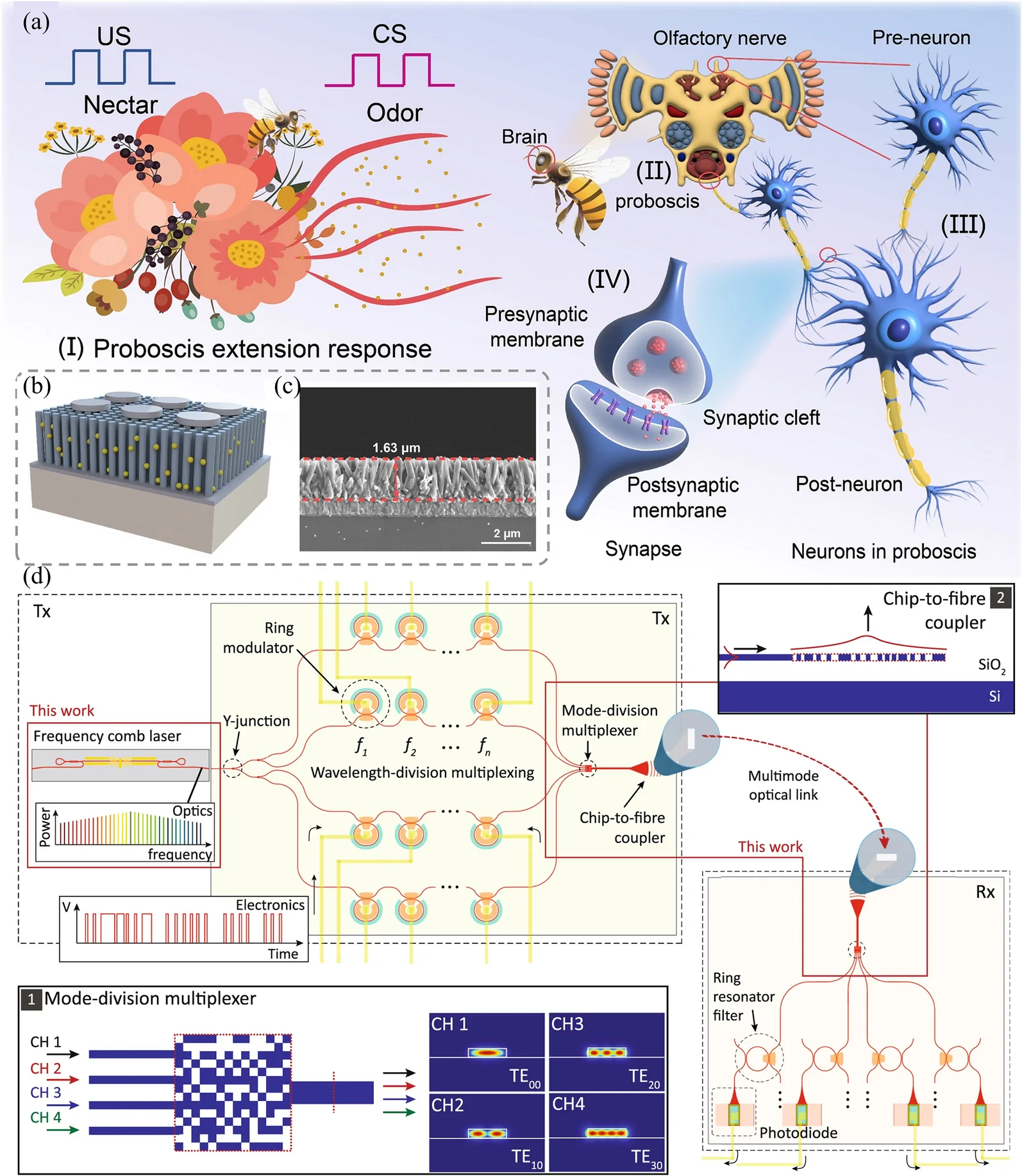
Schematic for the design strategies of AI chips in regard to data memory and transfer. **a** Schematic illustration of the proboscis extension response. **b** Schematic of the ATZ-based device. **c** SEM of the as-prepared device. **a**–**c** Reproduced with permission from Ref. [36] Copyright 2024, Springer. **d** Schematic illustration of the multi-dimensional communication. **d** Reproduced with permission from Ref. [59] Copyright 2022, Nature

The complexed and comprehensive simulations about the functions of the biological learning and memory are expected to be accomplished by the artificial neuromorphic devices [36]. A large amount of research has been launched focused on the neuromorphic electronics featured with massive parallelism, high efficiency, and capability. In particularly, as a form of associative learning, classical conditioning generally comprised of conditional stimuli (CS) and unconditioned stimuli (US) contains four features, including acquisition, extinction, recovery, and generalization, which are relevant to information storage, elimination of outdated information, rememorizing, and storage of new information in a cycle [114]. Accordingly, synaptic electronics equipped with associative learning capabilities are potential candidates for next-generation AI. Light has been used to coordinate with electrical devices to fully realize the aforementioned four features of classical conditional when taking the shortcomings of crosstalk, poor sustainability, and complex circuits for purely electrical signals with into account [36]. What is more, the difference in the aspect of relaxation times between and electrical stimuli and light endows the devices an inherent advantage to realize the characteristics of classic conditioning. The associative learning was accomplished by optoelectronic memristors based on Ag/TiO_2_ nanowires (NWs): ZnO quantum dots (QDs)/FTO (ATZ-based device). As is shown in Fig. 7a, the flower nectar was served as the US that caused the proboscis extension, while the flower odor was served as CS which must be trained through the coordination of the olfactory and proboscis nerves to trigger the proboscis extension directly. A two-port ATZ-based memristive device was designed to simulate the synaptic behavior with a structure of the vertical arrangement similar to that of the synapses (Fig. 7b), and the SEM of the as-prepared device is demonstrated in Fig. 7c. It was verified that in addition to the basic synaptic behaviors, more advanced synaptic functions like learning-forgetting-relearning functions could also be achieved.

### During Transmitted Process

The issue of data transfer limit for high-performance silicon chips has drawn a lot of attention, for which several schemes have been proposed [59]. Optical computing has great potential in improving the speed of a diversity of ML applications, which is attributed to its enhanced data transfer, low latency, and fast computation rate when taking the fact that light travels much faster than electrical signal under considerations [13, 23, 58, 115, 116]. Besides, the use of optical interconnects has become as a potential technology that can address this problem. It is pointed out that the chip-scale optical interconnects are promoted by the development of wavelength-division multiplexing (WDM) technique, which makes it possible to realize the parallel signal transmission by means of encoding data independently carried on multiple frequencies of light [117, 118]. After that, in order to further increase the link bandwidth, attentions have been paid on the other promising dimension of signal encoding for multiplexing, like the spatial domain. To be specific, the light can be decomposed into a series of optical beams with orthogonal spatial cross sections, and these orthogonal spatial modes can act as independent communication channels [119–126]. It is possible for each of them to support a full WDM link, leading to the multiplicative effect on the bandwidth of an optical link provided by the mode-division multiplexing (MDM). Latterly, progress has been made focused on the integrating mode and WDM on a chip [127–133].

In an attempt to offer new dimensions of data transfer with the aim of fulfilling the growing need for speed, an integrated multi-dimensional system that integrated wavelength and mode multiplexing on a silicon photonic circuit for the on-chip and chip-to-chip interconnects was put forward [59] (Fig. 7d). A multi-wavelength laser source was evenly distributed into multiple WDM transmitter circuits with each WDM circuit encoding data independently onto different frequencies of light. An inverse-designed MDM multiplexer took the overlapping modes from the multiple WDM transmitters, and after that they were transformed into copropagating spatially orthogonal modes. The data could then be transmitted through chip-to-fiber couplers and multimode fiber to the receiver. The MDM-WDM demultiplexers were used to separate the mode and wavelength channels, and photodiodes were taken advantages of for detection. It was verified that a 1.12-Tb/s natively errorfree data transmission could be fulfilled.

### At the Computing Stage

**Fig. 8:**
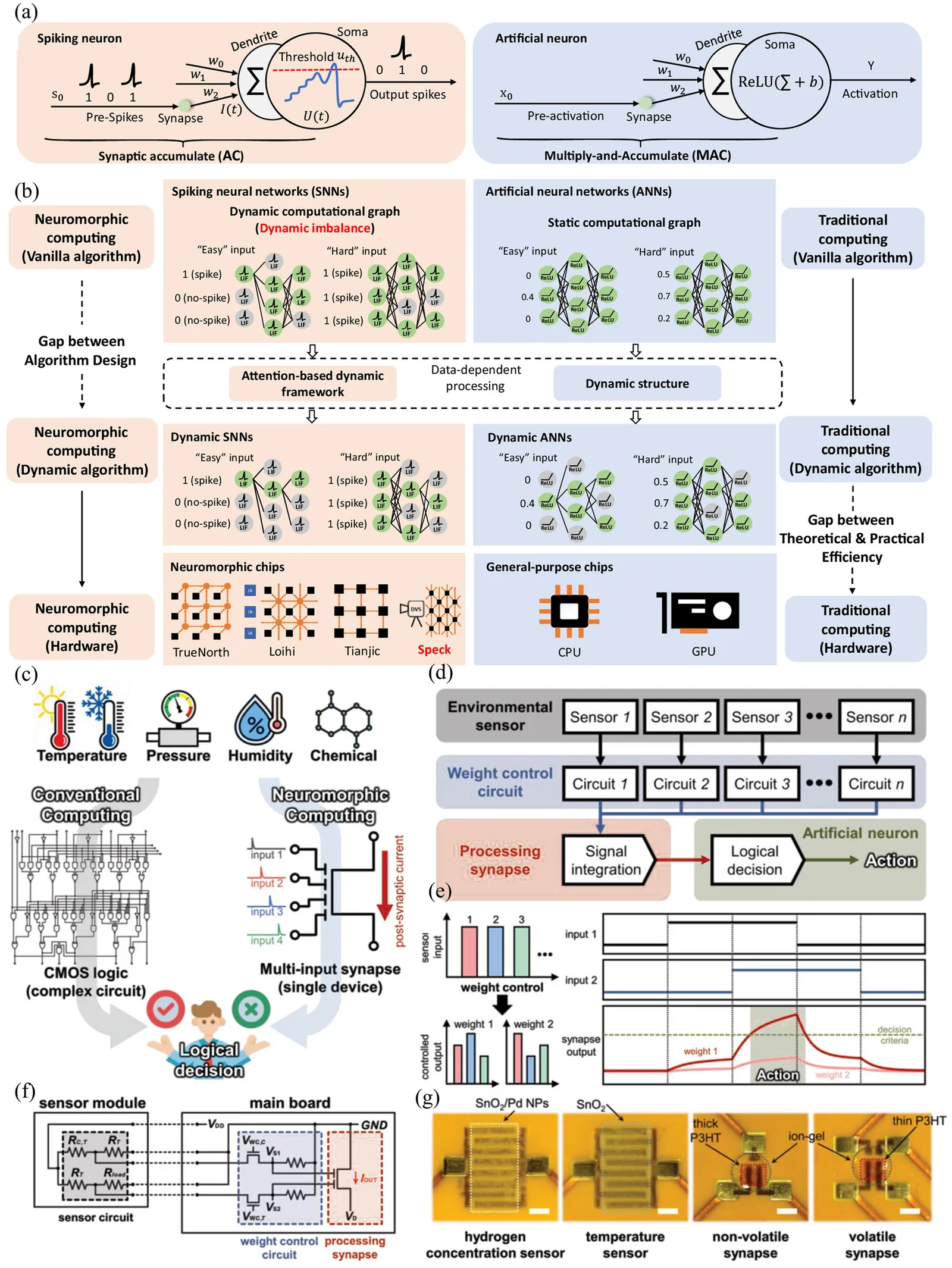
Schematic for the design strategies of AI chips in regard to computing. Comparison between **a** spiking neuron and artificial neuron, and **b** the neuromorphic and traditional computing for a dynamic computing. **a**, **b** Reproduced with permission from Ref. [37] Copyright 2024, Nature. **c** Comparison between conventional and neuromorphic computing. **d** Schematic illustration and **e** schematic signal flow of the neuromorphic signal integration system. **f** The circuit diagram and **g** photographic image of the hydrogen explosion risk assessment system. **c–g** Reproduced with permission from Ref [39]. Copyright 2024, Wiley–VCH GmbH

Dynamic computing is a promising approach in DL, and the dynamic neural networks are managed to adapt the computational graphs to the input in the inference stage, showing the attractive properties in many aspects [134]. The neuromorphic and traditional AI systems are two typical paradigms for dynamic computing [37]. Particularly, neurons in SNNs communicate through spike trains, and the spike-based neuromorphic computing is naturally featured with a dynamic computational graph, with only a small portion of the overall spiking neurons being active at any moment and the rest being idle. In contrast, the neurons in traditional Artificial Neural Networks (ANNs) exchange information via continuous values and are controlled by static computational graphs. As a result, dynamic algorithms are developed to implement dynamic computing (Fig. 8a, b).

The energy constraints become a major restriction to deploy traditional AI methods, and therefore high demand for the energy efficiency has also been proposed for the computing. Correspondingly, much efforts have been made to come up with the schemes for energy-efficient computing. For example, better energy efficiency can be offered by analog in-memory computing (analog-AI) as it can perform matrix–vector multiplications (MVM) in parallel on ‘memory tiles’ [1]. Besides, the neuromorphic computing provides a promising way for energy-efficient machine intelligence by learning from the way by which information is processed via brain, taking advantages of artificial neurons and the SNNs on neuromorphic chips. The neuromorphic computing meets the challenges of how to learn from the high-level brain dynamic mechanisms to realize the excellent computational efficiency [37].

In addition to the requirement from dynamic computing and energy constraints, high demand has also been put forward for the weight-reconfigurable capacity of computing for some fields, like the healthcare monitoring, on which occasion it is essential to finely reconfigure the relative intensity of weight from each input. In an attempt to achieve the precise and independent modification of each input, a neuromorphic computing system that was managed to integrate two different environmental information with reconfigurable weights by making use of a simple circuitry based on electrochemical artificial synapses was designed [39]. From the perspective of dealing with various environmental information, a complex logic circuit was essential with the increased complexity of the processor, since more environmental factors need to be taken into consideration for a conventional CMOS-based processor, while a single device was managed to handle these environmental information by neuromorphic computing with an electrolyte-based multi-input synapse, which is demonstrated in Fig. 8c. Schematic illustration of the neuromorphic signal integration system is shown in Fig. 8d. To be specific, the sensors were responsible for the transform of the raw data into electrical signals, and then a weight control circuit was made use of to assign weights to the signals. The processing synapse could then integrate the signals, and finally a logical decision could be made by the artificial neuron. Correspondingly, the schematic signal flow of this system is demonstrated in Fig. 8e. Action was executed if the synapse output exceeded the level of the criteria. It was noticeable that the potentiation of the processing synapse was modulated with the different weights for signals, leading to the different final action state even for the same environmental signals. A hydrogen explosion risk assessment system was designed accordingly, with the schematic circuit diagram shown in Fig. 8f and the photographic image demonstrated in Fig. 8g. Hydrogen concentration and temperature were used as the inputs, and the signals were then updated by the weight control circuit, after which procedure they were converted into a postsynaptic current to represent the hydrogen explosion risk by taken advantages of the multi-input artificial synapse.

## Design Considerations for Future Advanced and AI Chip

A sharply increased calculations have been brought about with the development of AI technology [39]. The prosperity of AI is largely empowered by a significant amount of parameters and improved computing powers [34]. As to many vision tasks, short exposure time is essential to complete the tasks with ultra-low latency, calling for extremely high computing power [27]. In addition, the computing capability and energy efficiency are critical issues which need to be balanced for high-performance computing [135].

### For High-Performance Computing

#### To Accelerate Computing Speed

**Fig. 9:**
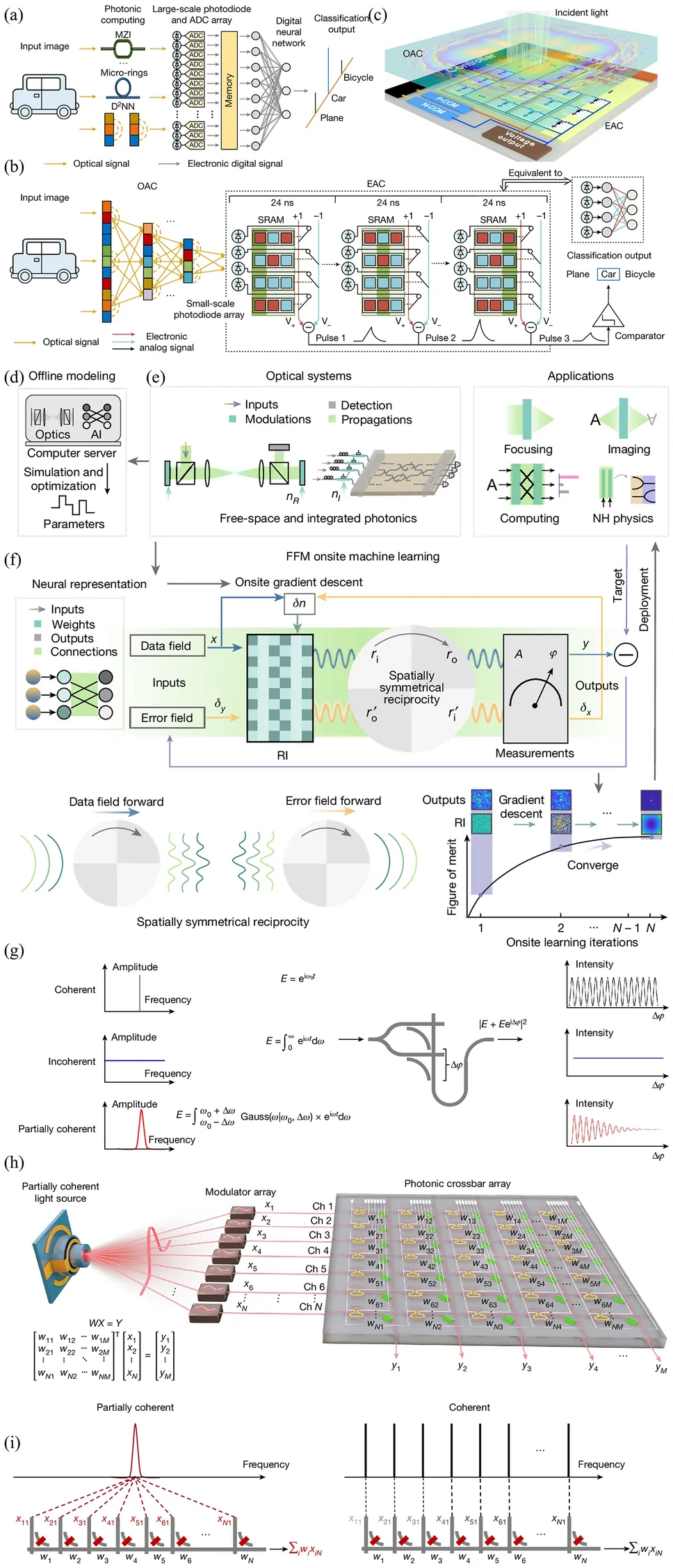
Design considerations of AI chips for high-performance computing. The workflow of **a** traditional optoelectronic computing, and **b** all-analog photoelectronic computing. **c** Schematic diagram of the all-analog photoelectronic chip. **a–c** Reproduced with permission from Ref. [27] Copyright 2023, Nature. **d** Schematic diagram for the conventional optics-related AI and **e** the general optical systems. **f** Schematic illustration of FFM onsite ML. **d–f** Reproduced with permission from Ref. [35] Copyright 2024, Nature. Schematic illustration of **g** a generalized unit cell with coherent light sources, and **h** the proposed photonic convolutional processing system with partially coherent light. **i** Schematic illustration for the *N*-fold enhancement in regard to parallelism. **g–i** Reproduced with permission from Ref. [43] Copyright 2024, Nature

The computing speed should be further accelerated to cooperate with the improved performance of various tasks at the algorithmic level [13, 136]. Large-bandwidth and high energy efficiency computing can be achieved by optical AI for which optics and photonics are fully leveraged. A fact that cannot be ignored is that digital devices remain to be the mainstream, and therefore it is essential to convert the optical signals into digital ones for vision tasks even after optical computing by means of large-scale photodiodes and power-hungry ADCs to conduct the necessary postprocessing procedures [27] (Fig. 9a). In an effort to address this issue, an optoelectronic hybrid architecture was designed, which was managed to reduce massive ADCs, and therefore vision tasks could be accomplished in a power-efficient and high-speed manner (Fig. 9b). To be specific, the information was encoded into light fields. The features of high-resolution images were extracted by using a multi-layer diffractive optical computing module at light speed, which was optical analog computing (OAC). It was worthwhile mentioning that the demand for optoelectronic conversion could be reduced by dimension reduction all optically. The electronic analog computing (EAC) with a 32 × 32 photodiode array was then introduced to convert optical signals into analog electronic ones due to the photoelectric effect, working as a nonlinear activation. These photodiodes are either connected to the V _+_ positive line or V_-_ negative line according to the weights in the static random-access memory (SRAM). Based on Kirchhoff’s law, the generated photocurrents were summed up on both lines, after which process the differential voltage of the computing lines V_+_ and V_-_ was calculated by the analog subtractor as the output node. It was noticeable that by means of resetting the computing lines and updating weights, this system can output another pulse with different connections of photodiodes. The output could be used either as predicted labels of classification categories or as inputs of another digital neural network. Schematic diagram of the all-analog photoelectronic chip is demonstrated as Fig. 9c.

Another challenge met by the optical computing is that they are implemented in silico on electronic computers, and therefore both strict modeling and large amounts of training data are essential (Fig. 9d). In particularly, optical AI primarily includes the optical emulation of electronic ANNs, and the photonic architecture design is conducted on electronic computers [24, 137]. Accordingly, it proposes the challenge of correcting the experimental system error which calls for extensive work to characterize the optical propagation spatially and temporally [83, 96, 98, 138]. As to AI empowered optical design, the system must also be modeled analytically or implicitly [139–141]. It consumes more time for analytical and numerical modeling with the increase of the system complexity. It is pointed out that the precise modeling of a general optical system is difficult to be achieved due to the system imperfections and the complexity of light-wave propagation. Some efforts have been made to address these issues [35]. FFM learning was developed, which mapped optical systems to parameterized onsite neural networks. It was worthwhile mentioning that by taking advantages of spatial symmetry and Lorentz reciprocity, the necessity of backward propagation in the gradient descent training was eliminated. Specifically, as for general optical systems, free-space lens optics and integrated photonics were contained, with the modulation regions marked as dark green and propagation regions demonstrated as light green, in which occasion the refractive indexes were respectively, tunable and fixed (Fig. 9e). These regions in the optical system could be mapped to weights and neuron connections, which made it possible to construct a differentiable onsite neural network between the input and output (Fig. 9f).

#### To Realize High-Capacity Signal Processing

In addition to the method mentioned above, parallel multi-thread processing is also one of the key approaches to achieve high-speed and high-capacity signal processing, which is a promising way to meet the increasing demand for high-capacity datasets processing [142]. Recently, a photonic convolutional processing system using partially coherent light to realize boost computing parallelism without substantially sacrificing the accuracy has been proposed [43]. It was pointed out that a variety of system architectures for photonic convolutional processing was developed with the coherent light sources being applied in all of these cases. However, the operation of the coherent nanophotonic circuits needed the precise control of numerous phase shifters so that the desired coherent interference in the circuit could be achieved. A generalized unit cell to perform multiply-and-accumulate operations is illustrated in Fig. 9g, while a system with partially coherent light for parallelized photonic computing is proposed as Fig. 9h. It was worthwhile mentioning that for the system with partially coherent light for parallelized photonic computing, the coherent light source was not necessary, leading to less rigorous feedback control and thermal-management requirements. As for the partially coherent system, a Gaussian-shaped optical carrier could be sent to all input channels and summed in a bus waveguide, while for a coherent system, different input channels should receive optical carriers at distinct wavelengths to avoid intensity fluctuation. As a result, one MVM operation for input vectors of dimension *N* called for only one optical band for partially coherent system, while *N* optical bands were required with coherent light being applied, making it possible for the *N*-fold enhancement in parallelism as using partially coherent light (Fig. 9i).

### With Improved Energy Efficiency

#### General Approaches to Improve the Energy Efficiency

In addition to the enhanced computing performance, the high energy efficiency is another important requirement for the advanced chips. For example, in regard to many vision tasks, the ADCs with high throughput and high precision reduce the imaging frame rate on account of limited data bandwidth, causing remarkable energy consumption [27]. Accordingly, efforts have been made on the design of an optoelectronic hybrid architecture in an all-analog way, to reduce the massive ADCs for the accomplishment of power-efficient vision tasks. Furthermore, neuromorphic computing tends to be a promising approach for energy-efficient machine intelligence by simulating the neurons of the human brain and using spiking neural networks [37]. It is proposed that the human brain is managed to allocate its resources dynamically according to the required demand [143, 144]. As a result, greater attention is paid to salient stimuli, which is proved via the heightened spiking activity of the brain regions or neurons associated with the stimulus. Additionally, endeavors have also been made to design the neuromorphic chip with no needs for the global or local clock signal, which efficiently avoids the redundant power consumed by the clock empty flips [37]. Furthermore, it is worthwhile mentioning that CIM is important in the field of AI, for which both the memory and processing functions can be integrated within the same module, leading to the enhanced efficiency. Memristors, which are featured with their striking similarity with biological counterparts in the aspect of device dynamics, play an important role in this field [145].

#### Analog In-memory Computing

**Fig. 10:**
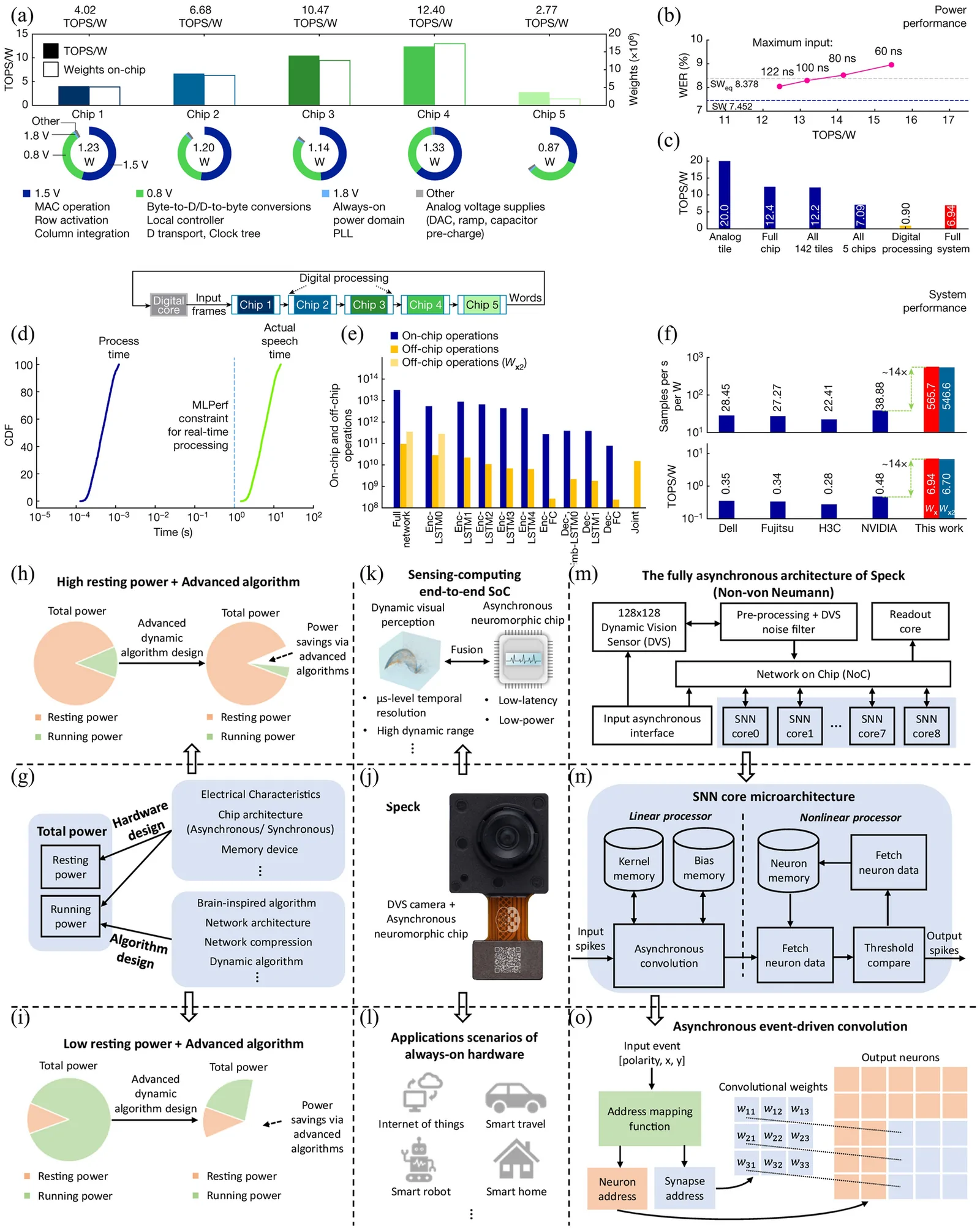
Design considerations of AI chips with improved energy efficiency. **a** Measured power and TOPS/W corresponding to each chip. **b** An improvement in TOPS/W caused by the reducing the maximum input duration. **c** Energy efficiency at different levels. **d** Processing time and actual speech time. **e** Number of operations performed on-chip versus off-chip in the RNNT experiment. **f** Samples per second per watt and TOPS/W compared with MLPerf submissions. **a**–**f** Reproduced with permission from Ref. [1] Copyright 2023, Nature. **g** Power composition of AI systems. The case of **h** high resting power and **i** low resting power. **j** Physical display of Speck. **k** Illustration for the sensing-computing end-to-end SoC, and **l** its application scenarios. **m** Fully asynchronous architecture of Speck. The design of **n** SNN core, and **o** the asynchronous event-driven convolution. **g**–**o** Reproduced with permission from Ref. [37] Copyright 2024, Nature

The vast amounts of data transferred between memory and processor lead to the unessential energy consumption. Both the time and the energy are expected to be saved by the Analog-AI hardware with the function to apply arrays of non-volatile memory (NVM) to execute the MAC operations. One case in point was that an analog-AI chip was designed to recognize and transcript speech energy efficiently. It was noticeable that not only the fully end-to-end SW_eq_ accuracy for a small keyword-spotting network but also the near-SW_eq_ accuracy on the much larger MLPerf RNNT was verified [1]. Particularly, the tiny-model task of keyword-spotting network (KWS) on the Google speech-commands dataset was targeted. The MLPerf version of RNNT, which was a large data center network, was implemented on Librispeech. It was worthwhile mentioning that the model contained 45 million weights, which was implemented by more than 140 million PCM devices across five chips. This system demonstrated excellent power performance. To be specific, Chip 4 showed the best power performance of 12.40 TOPS/W, which was attributed to the most on-chip weights (Fig. 10a). It was proposed that there existed a correlation between the reported TOPS/W and the number of weights that were encoded on-chip. Another 25% improvement in TOPS/W could be achieved for chip 4 caused by the reducing the maximum input duration without large WER degradation, which is illustrated in Fig. 10b. Energy efficiency at different levels is illustrated in Fig. 10c, which reflected how the costs of data communication, incomplete tile usage, as well as the inefficient digital computing resulted to the fact that the large peak TOPS/W of the analog tile itself was down to the final sustained value of 6.94 TOPS/W. The full processing time of the overall system was estimated (Fig. 10d). It was noticeable that the average processing time for each sample was more than 10^4^ times faster than the actual speech time, leading to a real-time factor of only 8 × 10^–5^. Number of operations performed on-chip versus off-chip in the RNNT experiment is shown in Fig. 10e. In contrast to the MLPerf submissions, a 14-fold improvement was managed to be realized by this system in regard to the samples per second per watt and TOPS/W (Fig. 10f).

#### Dynamic Computing with Asynchronous Chip

To reach the goal of energy efficiency, the composition of different power consumption should be taken into considerations. The power that is required to operate an AI system is usually composed of two aspects, resting power which is determined by the hardware design, and running power which relies on the model as the hardware is fixed [37] (Fig. 10g). It is proposed that for the great majority of hardware a significant amount of energy is consumed even when no computing is being done, leading to very high ratio of the resting power to the overall power. Consequently, it is difficult to reduce the overall power only by reducing the running power (Fig. 10h, i). To be specific, the chip architecture (asynchronous/synchronous) can leave an impact on the power consumption, and it has been proposed that the asynchronous architecture, for which the change of the circuit state is only caused by the change of the external input, is featured with the advantage of low power consumption compared with synchronous circuits. The event-driven mechanism is an approach for asynchronous chips to coordinate the work of each module. When taking the design strategies for sensing-computing chip into considerations, event-driven chips can be made use of, since the sensor can only wake up the chip when the environmental changes (such as temperature changes or motion triggers) are detected to complete data collection and transmission, leading to the improved energy efficiency and low latency.

In contrast to the most common neuromorphic hardware design which begins with the bottom of the compute stack, elaborated design can be conducted for the customization of the neuromorphic hardware which is to be applied at the edge for the specific purposes with low power consumption taken into consideration. One case in point was that a sensing-computing neuromorphic chip, Speck, was designed with a 128 × 128-pixel DVS integrated onto an asynchronous spike-based AI chip, which is shown in Fig. 10j. Speck was a sensing-computing end-to-end SoC with the always-on hardware applicable to various scenarios, such as Internet of things, smart travel, smart home, intelligent robotic, and so on (Fig. 10k, l). It was worthwhile mentioning that its processing pipeline was built with asynchronous digital logic, which made it possible for the chip to realize always-on low resting power consumption and optimum latency. To address the issue that the implementation of asynchronous circuits is complicated, the overall sensing to computing strategy was optimized. There was a central event router which is able to be configured to route events from any to any of the 9-SNN cores, and every core was managed to work independently and asynchronously, which was illustrated in Fig. 10m. As a result, the design effort could be limited to a single SNN core (Fig. 10n). Additionally, the asynchronous event-driven convolution was included as one of the core designs for the improvement of the computational efficiency as well (Fig. 10o).

## Perspectives

**Fig. 11:**
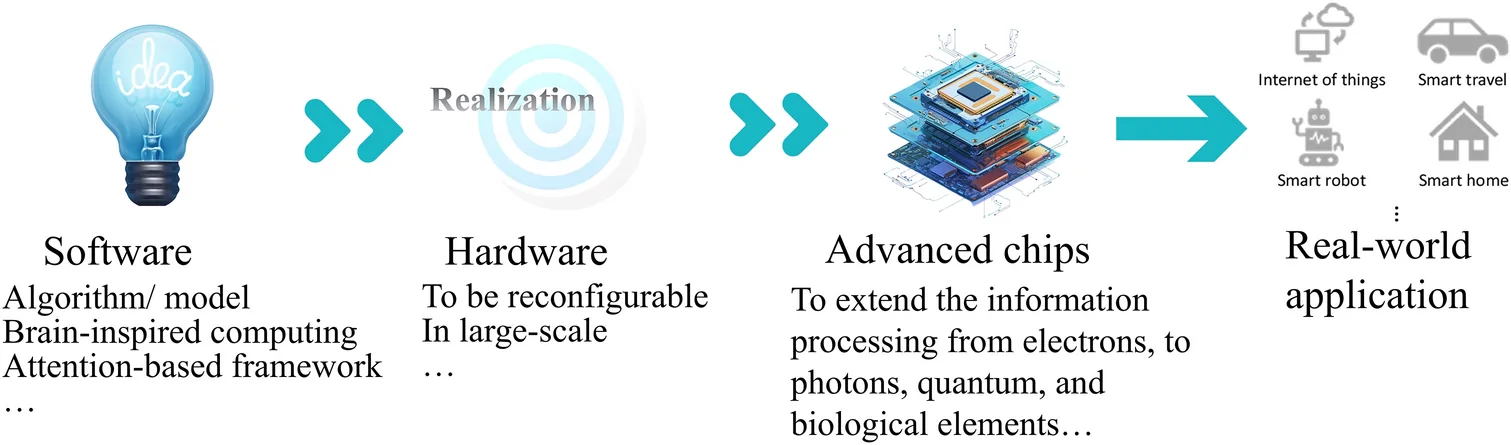
Outlook of the advanced chips

Overall, the recent development, including but not limited to the co-design strategies for the software and hardware, the realization of enhanced overall performance, and the potential for broader application have been reviewed in depth. Great progress has been made in the field of advanced chips due to the high challenges brought by AI, which has revolutionized various aspects, ranging from information industry to material science. To execute the complex algorithmic programs and advanced tasks proposed by these new challenges, the elaborate design of chips covers every aspect, including materials, algorithm, architectures, processing technology, integrating method, and so on. Progress has been made on developing novel materials and models, as well as overcoming the shortcomings of the existing conventional materials and architectures for chips. New fabrication processes for both the production and the package of the devices have been developed, aiming to induce the cost and develop complex chips. The advanced chips are qualified to be applied for video recognition tasks, speech recognition and transcription, visual memory and many other fields, offering fast and efficient information processing functions (Fig. 11).

Summary for the state-of-the-art advanced and AI chips is illustrated in Table 1 with the performance, scales, other properties, and applications included. The quantitative indicators of the chips are critical to the systems. To be specific, energy efficiency refers to the effective amount of work completed by a chip with per unit of energy consumed when implementing a task, which makes sense for the environmental sustainability. The computing speed of a chip is the core indicator for measuring its data processing capability, which is important for shortening the task processing time and supporting complex tasks. For AI training which needs to handle large amounts of parameters, chips with high computing speed are managed to shorten the training cycle, accelerating technological iteration. The latency of a chip refers to the time interval from the triggering of an input to the generation of an effective output, which is a key indicator for measuring the response speed of a chip. While ensuring high energy efficiency and computing speed, reducing latency has become another challenge in chip design, which is especially essential for some real-time tasks. Besides, the abilities of integrating more transistors, realizing a larger area, or expanding to more application fields are also imperative for these systems. For example, the scale expansion of chips is in relevant to the change from achieving a single function to multi-functions or from small-scale to large-scale applications, which can leave impacts on a series of factors, like cost, power consumption, design complexity, and so on.

**Table 1:** Summary for the performance of the state-of-the-art advanced chips

	Type	Energy efficiency (TOPS/W)	Computing speed (TOPS)	Latency	Scale	Accuracy	Other key features	Application	References
Neuromorphic chip	Analog-AI chip	12.4	–	2.4 μs for each audio frame	To combines 35 million phase-change memory devices across 34 tiles and	With fully end-to-end SWeq accuracy for a small keyword-spotting network	To show a 14-fold improvement compared with traditional ones, and to demonstrated a WER of 9.258%	Speech recognition and transcription	[1]
	Integration of trainable dendritic neurons and high-density RRAM chip	–	–	380 ns	–	~ 90%	To realize 516 × and 1.3 × 10^5^ × improvements on the LAE (LAE = Latency^−1^ × Area^−1^ × Energy^−1^) FoM when compared to digital and analog CMOS activation circuits	For CIM-based neuromorphic computing	[34]
	Neuromorphic hardware	–	–	–	A 3 × 7 memristor array	An accuracy of 88.9% for handwriting digit recognition	To realize complex biological associative learning behaviors	Visual memory application	[36]
	Sensing-computing neuromorphic chip	–	–	Less than 0.1 ms	To be an efficient medium-scale neuromorphic sensing-computing edge hardware	92%	With the low processor resting power of 0.42 mW and real-time power as low as 0.70 mW	As edge computing devices for smart home application scenarios	[37]
	Neuromorphic computing systems	–	–	–	–	–	To be weight-reconfigurable	For hydrogen explosion risk assessment	[39]
	Neuromorphic optoelectronic computing system	1.58	240.1	–	–	With a blind-testing accuracy of 97.6% on 10,000 digit images	To be reconfigurable	For high-speed image and video recognition	[83]
Photonic chip	Photonic computing	1	0.108		With a 3 × 3 photonic tensor core, using phase-change-material photonic memories	92.2% accuracy (92.7% theoretically)	To boost computing parallelism while maintaining the accuracy	To classify the gaits of ten patients with Parkinson’s disease with	[43]
	A silicon photonic circuit	–	–	–	Multimode optical transmission between separate silicon chips	–	With a 1.12-Tb/s natively errorfree data transmission	Silicon photonic transmitters	[59]
	A trainable diffractive optical neural network	7.28	217.6	30.2 ps	With 1 × 16 neurons input, 5 × 16 neurons hidden and 1 × 4 neurons output layers	85.7% accuracy for multimodal test sets	With high computing density (447.7 TOPS/mm^2^)	To accomplish four-class classification in different modalities	[41]
	Photonic chiplet	160	–	3.79 ms	With 4256 total neurons and a net scale of 13.96 million	Testing at 91.89% accuracy in the 1623-category Omniglot dataset	To experimentally achieve on-chip 1000-category-level classification and high-fidelity AI-generated content with up to two orders of magnitude of improvement in efficiency	For large-scale photonic computing and artificial general intelligence (AGI)	[38]
	Photonic convolutional accelerator	–	11.3	< 200 ps	–	With an accuracy of 88% for recognition of handwritten digit images	For generating convolutions of images with 250,000 pixels	For real-time video recognition	[22]
	An integrated photonic tensor core	0.4	–	–	With the matrix size being easily be scaled up to 40 × 40	With an accuracy of 95.3%	With computing densities of more than 400 TOPS per mm^2^	For parallel convolutional processing	[24]
	An on-chip photonic DNN	–	0.27	570 ps	To be scaled to a classifier with a larger number of pixels	With an accuracy of 93.8% for two-class classification of handwritten letters	With a classification time of under 570 ps	For image classification	[25]
	Photonic processing unit	0.2	–	–	–	With the accuracy 96.6% of recognition	With a preeminent photonic-core compute density of over 1 TOPS mm^−2^	For image reconstruction, video action recognition, and autonomous driving	[149]
	All-analog photoelectronic chip	7.48 × 10^4^	4.6 × 10^3^	72 ns for each frame	With two 400 × 400 SiO_2_ OAC layers and a 1,024 × 3 EAC layer	92.6% for time-lapse video recognition task	With superior system robustness in lowlight conditions (0.14 fJ μm-^2^ each frame)	Time-lapse video recognition task	[27]
	All-optical processing	5.40 × 10^6^	–	–	–	94.5%	To facilitate orders-of-magnitude-faster learning processes	To design non-conventional imaging modalities	[35]
	Chip integrated meta surfaces	–	–	–	–	–	With the potential to be compatible with on-chip optical systems and to independently encode multiple optical parameters	For multidimensional encryption	[150]
Quantum chip	Quantum simulator	–	–	–	–	–	To realize the stable trapping of 512 ions in a 2D Wigner crystal	To run noisy intermediate-scale quantum algorithms	[151]
Silicon-based chip	Biomimetic olfactory chips	–	–	–	With 10,000 individually addressable sensors per chip	With a prediction accuracy of up to 99.04%	With distinguishability of mixed gases and 24 distinct odors	To be integrated with vision sensors on a robot dog	[152]
	Si‑based optical memristive crossbar array	–	–	–	With a 5 × 5 optoelectronic synapse array	With a classification accuracy of 98.02%	To enables an ultralow power (2.8 × 10–^13^ J) fine-tuning process	For patient-specific issues	[153]

Significant improvements of the advanced chips have happened and accompanied by the discovery of novel modes, the improvement of the package techniques, the accelerating of the efficiency, as well as the enhancement of computing power. This review offers a keen insight into the design strategies for the advanced and AI chips, with some perspectives for the chips applied in the future proposed as follows:

1. Endeavors have been made to equip the AI chips with more intelligent performance learning from biology. a) Efforts have been made focused on mapping the biological behavior to the electrical behavior in devices. It is expected for the systems to realize more complex biological performances. The associative learning behavior, which is commonly found in the cranial nerves of insects and is featured with the acquisition, extinction, restoration, and generalization, has been simulated by ZnO QDs‑based optoelectronic memristors, which provide novel scheme for the field of machine self-learning. It is desirable to develop chips learning from more advanced behaviors of the creatures. b) Extensive investigations have been carried out on neuromorphic devices based on the human brain, which is a potential candidate for the next-generation computer architecture. The method of how to learn from the high-level brain dynamic mechanisms to equip neuromorphic computing with more energy advantages is always in high demand. Endeavors have been made from both the software and the hardware aspects to address this issue. Moreover, chips used for dealing with image information are expected to be managed to handle the dynamic, diverse, and unpredictable scenes in real application scenarios, like autonomous driving. It is desirable to design the chips that are efficient in various fields to percept and address even the difficult issues existing in the real world. In particular, the dynamic computing, which is a critical feature of human brain, has been simulated by this system. In the future, more advanced strategies can be adopted for the realization of high-level brain dynamic mechanisms to fully achieve the brain advantages in many aspects.
2. Efforts can be made to make full use of the novel modes that extend the information processing from electrons, to photons, quantum, and biological elements, by taking advantages of the strengths and overcoming their weaknesses. a) Photonics-based systems are managed to provide high-speed computing units, and therefore efforts have been made focused on the algorithms design to exploit their unique advantages. For instance, approaches have been developed to realize the high throughput and precision by the successful application of cellular automata [146]. Ultrafast silicon photonic reservoir computing engine has been developed, which paves the way for high-speed photonic computing [147]. For photonic computing, to truly become a leading technology in the field of AI, a series of key challenges still need to be meet which mainly lies in the aspect of integration, dynamic reconfiguration capability, standardization, and cost issues. In particular, the compatibility of silicon-based photonic chips with the existing CMOS processes needs to be optimized, and the capacity of photonic chips to dynamically adapt to different tasks is expected, since the hardware of photonic chips is relatively fixed. b) Low power consumption and real-time requirements have promoted the application of CIM in many fields, like intelligent sensors and IoT. For example, some progress has been made for cryogenic in-Memory Computing recently [148]. In the future, more endeavors can be made to enhance the computing abilities of the memory by making use of new materials, such as two-dimensional materials and oxide semiconductors, and optimizing the circuit architectures. Besides, 3D packaging can also be applied for CIM to obtain the systems with excellent overall performance. c) Additionally, cellular computing has emerged focused on the analysis and modeling of real cellular processes to implement computing with the aspects of information processing and adaptation. Attempt has been made on the reprogrammable circuits that are managed to increase circuit flexibility and realize the scalability of complex cell-based computing devices. The feasibility of proposing several circuits by making use of only a small set of engineered cells that can be externally reprogrammed to implement simple logics in response to the specific inputs has successfully been proved. In the future, more efforts can be made focused on taking advantages of biological circuits to implement logics and meet numerous biological challenges.
3. The advanced chips that are qualified for real-world applications are always in high demand. Multi-input signals are usually needed to be processed properly by the advanced processors suitable for diverse external information in the open-world applications. The integrated signals from different input are needed to be handled accurately and timely. The version of GPT-4 has successfully accomplished the processing of multimodal data, like images and audio. A neuromorphic computing system applied for the risk assessment has been developed with several kinds of factors taking into considerations. In the future work, more work focused in the development of algorithms and hardware tailored for open-world applications can be conducted. The overall performances are expected to be enhanced for the chips to meet the high requirement proposed by the real-world applications.
4. The reconfigurable behavior is an important aim for computing hardware. For the chips with reconfigurable capacities, their function can be changed even after the accomplishment of the fabrication, and therefore multi-modal data and different tasks can be dealt with, making the high flexibility in adapting to different tasks feasible. It is especially critical to the chips used for some specific purposes like healthcare monitoring, for which it is imperative to finely reconfigure the relative intensity of weight updates from each input. Explorations have been made to equip different types of chips with strong reconfigurability. The reconfigurability and multimodal capability have been achieved for a TDONN chip by taking advantages of on-chip diffractive optics with massive tunable elements. The reconfigurability has also been available for the diffractive-interference hybrid photonic chiplet, which is acted as the fundamental building block for a diversity of advanced ML tasks, with 1000-category classification and content generation included. An all-analog chip combining electronic and light computing (ACCEL) is also equipped with the reconfigurability for different tasks without changing the OAC module. The integration of two different information with reconfigurable weights has been accomplished by a neuromorphic computing system. In the future, the high degree of adaptability to different assignments empowered by reconfiguration is expected to be accessible for more chiplet when it is necessary.
5. More explorations on large-scale integrations are expected to be made for chips. With the increasing of information, chips are required to be integrated to an ever-growing level to process the booming signals. The large-scale integrations of various chips are indispensable to getting rid of the shortcomings of each chip. For inorganic counterparts, like CMOS chips, an integration level in ultra-large-scale has been realized, while poor mechanical compatibility with organisms exists. It is ideal for the devices to overcome inherent shortcomings and accomplish the large-scale integration. Moreover, the integrations are closely related to the technologies. A diversity of techniques like photolithography, screening, printing, and shadow-mask evaporation has been developed. In the future, the continuous progress of the techniques is expected to be made in order to miniaturize these devices.
6. The application of sustainable materials in AI chips is one of the most important trends in this field with the aim of reducing the environmental impact and improving energy efficiency. Efforts can be made from various aspects, such as selecting degradable substrates, developing environmentally friendly manufacturing process, preparing environmentally friendly heat dissipation materials, and so on. Some bio-elastomers with active-controllable degradation rates have been designed, which can be applied as the bio-electronic substrates and encapsulation layers. In the future, more endeavors can be made to make a balance between meeting the high-performance requirements of AI chips and controlling the costs when using sustainable materials.
